# Eosinophilic Esophagitis and Microbiota: State of the Art

**DOI:** 10.3389/fimmu.2021.595762

**Published:** 2021-02-19

**Authors:** Maurizio Mennini, Renato Tambucci, Carla Riccardi, Francesca Rea, Paola De Angelis, Alessandro Fiocchi, Amal Assa’ad

**Affiliations:** ^1^ Division of Allergy, Bambino Gesù Children’s Hospital-Istituto di Ricovero e Cura a Carattere Scientifico (IRCCS), Rome, Italy; ^2^ Digestive Endoscopy and Surgery Unit, Bambino Gesù Children’s Hospital-IRCCS, Rome, Italy; ^3^ Division of Allergy and Immunology, Cincinnati Children’s Hospital Medical Center, Cincinnati, OH, United States

**Keywords:** microbiota, eosinophilic esophagitis, probiotics, dysbiosis, toll-like receptor

## Abstract

Eosinophilic esophagitis (EoE) is a chronic, food-triggered, immune-mediated disease of the oesophagus, clinically characterized by symptoms referred to oesophagal dysfunction, and histologically defined by an eosinophil productive inflammation of the oesophagal mucosa, among other cell types. The involvement of an adaptive Th2-type response to food antigens in EoE was known since 2000; several cytokines and chemokines promote food-specific responses, during which local production of IgE, but also IgG4 derived from plasma cells in lamina propria of oesophagal mucosa might play an important role. Evidence pointing towards a possible role for the innate immunity in EoE has arisen recently. Together, this evidence gives rise to a potential role that the innate immune system in general, and also the microbial pattern recognition receptors (PRRs) might play in EoE pathogenesis. Among PRRs, Toll-like receptors (TLRs) are type-I transmembrane receptors expressed both on epithelial and lamina propria cells with the capacity to distinguish between pathogen and commensal microbes. As TLRs in the different intestinal epithelia represent the primary mechanism of epithelial recognition of bacteria, this evidence underlines that oesophagal TLR-dependent signaling pathways in EoE support the potential implication of microbiota and the innate immune system in the pathogenesis of this disease. The oesophagal mucosa hosts a resident microbiota, although in a smaller population as compared with other districts of the gastrointestinal tract. Few studies have focused on the composition of the microbiota of the normal oesophagus alone. Still, additional information has come from studies investigating the oesophagal microbiota in disease and including healthy patients as controls. Our review aims to describe all the evidence on the oesophagal and intestinal microbiota in patients with EoE to identify the specific features of dysbiosis in this condition.

## Microbiota in Eosinophilic Esophagitis: A Revision of Hygiene Hypothesis

Eosinophilic esophagitis (EoE) is a chronic immune-mediated disease of the oesophagus, characterized by oesophageal dysfunction and by a productive eosinophil inflammation of the oesophageal mucosa (1–3). The incidence of EoE has increased in recent years (4).

The involvement of an adaptive Th2-type response to food antigens in EoE is well demonstrated (5, 6); several cytokines and chemokines promote food-specific responses (7, 8), during which local production of IgE (9), but also IgG4 in lamina propria of oesophageal mucosa (10) may play an important role. Pro-fibrogenic factors released by inflammatory cells can provoke fibrous remodeling of the oesophageal mucosa (11, 12). Avoiding specific food triggers is, in some contexts, the first line of therapy for EoE (13, 14).

Rather than the specific adaptive immunity, the innate immune system recognizes and reacts to ecological insults and microbes without the need for an immunoglobulin-driven antigen-specific reaction. Proof pointing towards a possible role for the innate immunity in EoE has emerged. Oesophageal epithelial cells appear to be critical effectors provoking the inflammatory phenomena in EoE, not directly through eotaxin-3 release and other chemoattractants for eosinophils (15), but also by the recruitment of invariant natural killer T (iNKT) cells toward the oesophageal epithelium (16), which constitutes a crucial cytokine source. A pivotal role for mast cells (MCs) has also been recognized in the pathophysiology and symptoms of EoE, which reverse after effective dietary treatment (17). This evidence gives rise to a possible role that the innate immune system and raises some possible questions regarding the role of the microbial pattern recognition receptors (PRRs) in EoE pathogenesis.

Among PRRs, Toll-like receptors (TLRs) are type-I transmembrane receptors expressed both on epithelial and lamina propria cells with the ability to recognize microorganism and commensal organisms (18). In humans, there is an aggregate of 11 distinctive TLR, each having various specificities which, when activated, promote intracellular sign transduction pathways intervened by MAP kinases and NF-κB, at last setting off a supportive of inflammatory reaction. TLRs initiation is mindful, among different capacities, for setting off provocative reactions by going about as a connection among adaptive and innate immunity (19–21). Enactment and development of antigen-presenting cells and regulatory T cells (Tregs) rely in part upon TLR-intervened flagging, featuring their job on mucosal resistant homeostasis. A few investigations have assessed the association between hypersensitivity and TLR activation (19, 22, 23). TLR activity in oesophageal epithelial samples has been described (24).

Arias et al. shown that bacterial load and TLR1, TLR2, TLR4, and TLR9 were overexpressed on oesophageal biopsies with EoE compared to controls. Muc1 and Muc5B genes were downregulated while Muc4 was overexpressed. Upregulation of MyD88 and NFκB was discovered along with IL-1β, IL-6, IL-8, and IL-10 and PER-1, iNOS, and GRZA effectors. NG-K2D (KLRK1, IL-15, MICB) were likewise upregulated. In all cases, changes in EoE were neutralized after six food elimination diet (SFED) and mucosal healing.

As TLRs in the different intestinal epithelia represent the essential instrument of epithelial recognition of microbes (25), this proof underlines that oesophageal TLR-subordinate flagging pathways in EoE support the implication of microbiota and the innate immune system in the advancement of this condition.

Sterility in infant mice can prompt a move in the IgE-basophil axis, an unevenness in Th1/Th2 activation, just as inappropriate implication of Tregs. Human examinations to assess these components are not many (26, 27). These varieties may be suggestive of a connection between commensal microscopic organisms and hereditary demeanor to atopic illness. These bacterial ligands additionally could represent therapeutic targets in atopic disease.

Therefore, it appears potentially useful to understand the function of microbial flora in healthy human beings and the specific alterations in atopic diseases.

The White House Office of Science and Technology declared that the Fact Sheet for the National Microbiome Initiative (NMI) was intended to satisfy three explicit objectives: (1) to address central inquiries concerning the microbiome in different biological systems; (2) to create stage innovations for upgrading information sharing on microbiomes; and (3) to grow the microbiome workforce.

Since then, the interest in microbiome composition in different allergic conditions has grown (28–32).

The examination concerning how the microbiome can change, even in healthy individuals, is critical to improve comprehension of how the microbiome unthinkingly causes infection.

## Oesophageal Microbiota in Health

### Introduction Comments

Once thought composed of few microbes, the oesophageal mucosa showed a composition of around 300 bacteria species. New culture-independent techniques have allowed scientists to identify the microbial composition of the oesophagus.

The oesophageal mucosa hosts a resident microbiota, although in a smaller population as compared with other districts of the gut. The human microbiota of the digestive tract exhibits considerable qualitative and quantitative differences, with communities starting from 10 cells per g/mL of sampled material within the oesophagus and stomach to 10^12^ per g/mL of tested material in the large intestine (33, 34).

### The Main Findings

It was initially not clear whether the oesophagus was characterized by a defined microbiota. The first studies on the oesophageal microbiota and based on cultivation methods demonstrated that the oesophagus did not merely contain a transient microbial population originating from the oral cavity by swallowing or from the stomach by gastroesophageal reflux (GER) (35–37). It was later observed that bacteria were associated with the oesophageal mucosal surface, confirming the presence of a resident microbiota at this site (38). Knowledge regarding the composition of the microbiota of healthy individuals has been expanded using investigations based on metagenomics approaches (39–41).

In general, the distal oesophageal microbiota was described as simply like that of the oropharynx, yet not indistinguishable (38, 42, 43).

Scarcely any examinations have concentrated on the organization of the microbiota of healthy oesophagus, (44–46) but extra data has originated from considers researching the oesophageal microbiota in disease compared to controls (47–56).

### Influencers and Limitations

The accessible data on the microbiota are biased by various methodologies, contrasts in the tested parts of the oesophagus, and the heterogeneity of consideration/avoidance rules utilized in the different investigations don’t permit comparisons and make it hard to agree on the general microbiota synthesis of the healthy oesophagus.

The presence of Streptococcus spp. was described by all studies: along these lines, members from this genus, seem to be a dominant taxon in the microbiota of the healthy oesophagus. Other bacterial genera frequently identified in association with streptococci, albeit in lower extents, incorporate Fusobacterium, Veillonella and Prevotella. The nearness of different genera (e.g., Neisseria, Haemophilus, Gemella, Granulicatella, Actinomyces, Lactobacillus, Bacteroides, Porphyromonas, and Staphylococcus), was lower.

Outstandingly, the commonness of Fusobacterium, Streptococcus, Prevotella and Veillonella has reliably been accounted in several studies based on either culture-dependent or culture-independent methodologies and on various examples (biopsies, aspirates, brushes), subsequently giving a reliable sign of their commitment to the piece of the microbiota that colonizes the healthy oesophageal mucosa. The strength of streptococci and the continuous nearness of other taxa regular of the oropharyngeal microbiota have been identified with the piece of the microbial networks of the oropharyngeal cavity, where a high commonness of streptococci is discovered, along with Gemella, Fusobacterium, Veillonella, Rothia and Granulicatella, (57, 58) have bolstered the idea that the oesophageal microbiota is basically of oral inception.

In any case, not every single oral bacterium can colonize the oesophageal mucosa, while a few individuals from the oesophageal microbiota appear to be underrepresented in the oral cavity, highlighting an alternate microbiota variation in the two-body destinations (38, 45, 46).

## Oesophageal Microbiome and Diet

There is no sufficient data on the effect of the diet on the oesophageal microbiota. A study of 47 patients showed that a diet higher in fiber was associated with a decrease of Proteobacteria and an increase of the Firmicutes phylum in the oesophagus (59). Additional studies correlating oesophageal microbiome and nutrition are needed. Studies that further define the stability of the oesophageal microbiome over time as well as other factors that determine inter-individual microbiome composition will aid in our understanding of the role of the diet.

## Oesophageal Microbiota in EoE

### The Main Findings

In 2015, Benitez et al. described the bacterial composition of the oral and oesophageal mucosa through 16S rRNA assessment of buccal swabs and oesophageal biopsies from 33 pediatric EoE subjects compared to 35 non-EoE healthy controls. They applied a longitudinal model before and after defined dietary changes.

In this study, Firmicutes were more abundant in oesophageal compared to oral samples, and oesophageal microbiota was more abundant of Proteobacteria in controls than EoE. The authors detected a significant difference between activated EoE and controls biopsies. The targeted dietary intervention did not produce substantial differences in either oesophageal or oral microbiota; the reintroduction of allergens led to enrichment in Campylobacter and Ganulicatella genera in the oesophagus (55).

Corynebacterium was enriched in the EoE samples, as was Neisseria genus, as previously described in different inflammatory conditions (60, 61). The Atopobium and Streptococcus genera were consistently enriched in non-EoE control samples.

The oesophageal microbiome of non-EoE control subjects showed a prevalence of Gram (+) bacteria of the Streptococcus genus, in agreement with previous findings in adult (62) and pediatric individuals (45) without oesophageal inflammation. Most of Benitez et al. study subjects were pediatric males with nearly 100% documented and concurrent proton-pump inhibitors (PPI) use, providing a further indication of the resilience of the Streptococcus-dominated microbiome in the healthy oesophagus in all genders and age groups.

They did not detect differences in the oral microbiome between inactive EoE, active EoE or non-EoE control samples, suggesting that in pediatric EoE, bacterial communities are stable and might not be altered by dietary modification. Data do not support the use of oral samples for EoE surveillance instead of biopsies.

In the same year, Harris et al. performed a prospective evaluation of secretions from EoE adult and children oesophageal biopsies, Gastro-oesophageal Reflux Disease (GERD) and healthy mucosa through Esophageal String Test (EST). Bacterial load was determined by quantitative PCR. Bacterial communities, determined by 16S rRNA and 454 pyrosequencing, were compared between disease and health. The bacterial amount was increased in both GERD and EoE and compared to healthy subjects. In EoE individuals, the amount was increased regardless of treatment status or level of mucosal eosinophilia. Haemophilus was significantly increased in untreated EoE individuals as compared with healthy subjects. Streptococcus was diminished in GERD subjects in PPI therapy as compared with healthy subjects. These data affirmed that diseases related to mucosal eosinophilia are characterized by a different microbiome from that found in the ordinary mucosa (56).

The EoE activity did not seem to directly affect a load of bacteria in EoE. However, eosinophils possess extracellular DNA traps and numerous anti-microbial properties with the release of defensins (63–65). The microbiota of untreated EoE subjects showed a shift from a mostly Gram (+) population to an increase in Gram (−) bacteria similar to what has been described in GERD (49). The implication of Gram (−) bacterial involvement in reflux esophagitis (66) is consistent with the observed increase in Haemophilus and Proteobacteria in EoE. These data suggest that treatment could affect the microbiota.

Norder Grusell et al. enrolled 17 subjects with GERD and 10 with EoE. All patients performed endoscopic brush sampling and biopsies from the upper and lower oesophagus and the oral cavity. Bacterial growth was identified to the species or genus level. The major part of bacterial groups or species was found in specimens from the lower oesophagus in EoE subjects compared to GERD subjects. *Streptococcus viridans* was the most common bacteria in both groups. GERD individuals had significantly inferior bacterial diversity in both oesophageal and oral samples. This discrepancy could depend on the protective mucosal biofilm by the acid content in GERD patients. The authors endorsed cultural method and speculated that bacteria identified by 16SrDNA/RNA techniques might not be alive, and there might be amplification bias following the processing steps. Nevertheless, they acknowledge that it is impossible to cultivate some bacterial species, and essential bacteria might, therefore, be overlooked (67).

**Table 1** summarizes the main findings about microbiota in EoE.

**Table 1:** Main findings about oesophageal Microbiota in EoE.

Author, year	Study population	Method	Microbial differences
Benitez AJ. et al, 2015 (55)	Non-EoE pediatric controls and pediatric EoE subjects before and after defined dietary changes.	Bacterial composition through 16S rRNA gene sequencing of the oral and esophageal microenvironments using oral swabs and esophageal biopsies	1) Enrichment of Proteobacteria (Neisseria and Corynebacterium) in the EoE cohort, and predominance of the Firmicutes in non-EoE control subjects. 2) Targeted dietary intervention did not lead to significant differences in either oral or esophageal microbiota, reintroduction of highly allergenic foods led to enrichment in Ganulicatella and Campylobacter genera in the esophagus.
Harris JK. et al., 2015 (56)	EoE adult and children, Gastro-oesophageal Reflux Disease and healthy mucosa.	Bacterial composition of secretions and biopsies through 16S rRNA gene amplification from samples obtained with Esophageal String Test (EST). Bacterial load was determined by quantitative PCR.	1) In EoE, bacterial load was increased regardless of treatment status or degree of mucosal eosinophilia compared with normal. 2) Haemophilus was significantly increased in untreated EoE subjects
Norder Grussell E. et al., 2018 (67)	Subjects diagnosed with GERD and with EoE	Brush sampling and biopsies from the oral cavity, upper and lower esophagus. The samples were cultivated on agar plates, and bacterial growth was identified to the genus or species level and semi-quantified.	1) Significantly higher numbers of bacterial groups or species were found in specimens from the lower esophagus in subjects with EoE compared to subjects with GERD. 2) Streptococci were present in all of the EoE-subjects but only in approximately 75% in lower esophagus of the GERD-subjects, regardless of the sampling method.
Hiremath G. et al., 2019 (68)	Non-EoE pediatric controls and pediatric EoE.	The salivary microbiome was determined through 16S rRNA gene sequencing.	1) A trend toward lower microbial richness and alpha diversity was noted in children with EoE. 2) Specific taxa such as Streptococcus tended to be abundant in children with active EoE compared with non-EoE controls. 3) Haemophilus was significantly abundant in children with active EoE compared with inactive EoE and increased with disease activity.

## Helicobacter Pylori: A Controversial Role

Helicobacter pylori infection often occurs in early childhood and it seems to enhance immune-tolerance driving immune-mediated diseases in a susceptible host (69–71).

In this context, previous or current infection with Helicobacter pylori (exposure) has been reported to protect against EoE, perhaps owing to H. pylori-induced immunomodulation. In 2019 a meta-analysis evaluated 11 observational studies comprising data on 377,795 individuals worldwide. H. pylori exposure vs non-exposure was associated with a 37% reduction in odds of EoE (odds ratio, 0.63; 95% CI, 0.51–0.78) and a 38% reduction in odds of esophageal eosinophilia (odds ratio, 0.62; 95% CI, 0.52–0.76). Fewer prospective studies found a significant association between H. pylori exposure and EoE (p= .06) than retrospective studies. Effect estimates were not affected by study location, whether the studies were performed in pediatric or adult populations, time period, or prevalence of H. pylori in the study population (72). The role of H. pylori would therefore deserve more evidence.

## Possible Alternatives to the Oesophageal Biopsies for Microbiota Evaluation

In 2012 Fillon et al. described the oesophageal microbiome in healthy children through the Enterotest™ (EST), a minimally invasive string technology. EST samples and mucosal biopsies were collected from healthy children (n=15) and their microbiome composition determined by 16S rRNA gene sequencing. Microbiota from oesophageal biopsies and ESTs produced nearly identical profiles of bacterial genera and were different from the bacterial contents of oral and nasal cavity samples. They concluded that the EST could be a useful device for the study of the oesophageal microbiome (45).

In 2019 Hiremath et al. collected saliva samples from 19 non-EoE controls and 26 children with EoE. The salivary microbiome was determined through 16S rRNA gene sequencing, and disease activity was assessed through the Eosinophilic Esophagitis Histologic Scoring System (EoEHSS) and the Eosinophilic Esophagitis Endoscopic Reference Score.

A trend toward lower microbial richness was recognized in EoE children. The salivary microbiome was similar between children with and without EoE.

Streptococcus tended to be more abundant in children with active EoE compared with non-EoE controls. Haemophilus was significantly plentiful in active EoE compared with inactive EoE and increased with the increasing Eosinophilic Esophagitis Histology Scoring System and EoEHSS. Besides, four broad salivary microbial communities correlated with the EoEHSS.

The authors concluded that the composition of the salivary microbiome community structure could be different in children with EoE. The disease activity positively correlates with the relative abundance of Haemophilus. Perturbations in the salivary microbiome may play a role in EoE pathobiology and could be a noninvasive marker of disease activity. The disease activity in the oesophagus does not seem to affect a load of bacteria in EoE directly (68).

## Attempts at the Modulation of EoE with Probiotic

Recently, it was demonstrated that there are huge contrasts in gut microbial community structure, microbial abundance, and uniformity in patients with EoE.

Faecal microbiota was evaluated through 16SrRNA amplification from 12 EoE patients and 12 controls. Patients with EoE showed inferior gut microbiota alpha diversity. The authors observed at the phylum level an important increase in Bacteroidetes and a decrease in Firmicutes and a significant reduction in Clostridiales and Clostridia at the order and family level in patients with EoE.

The authors speculated that Clostridia based interventions could be tested as adjuncts to current therapeutic strategies in EoE (73).

The probiotic Lactococcus lactis NCC 2287 has previously been shown to decrease clinical scores in a food allergy model based on co-administration of cholera toxin and ovalbumin (74). Also, NCC 2287 is a potent inhibitor of the eosinophil survival cytokine IL-5 and an inducer of the immune-modulatory cytokine IL-10 and in Th2-skewed cultures of peripheral blood mononuclear cells (PBMC) (75). The probiotic Bifidobacterium lactis NCC 2818 is also known for its immunomodulatory properties in allergy (76).

Holvoet et al. in 2016 tested L. lactis NCC 2287 and B. lactis NCC 2818, for their capacity to decrease oesophageal inflammation in EoE murine model (77).

To test whether probiotics could decrease oesophageal eosinophilia in an EoE animal model, the strain NCC 2287 was added to the drinking water as prevention (day 0 to day 28; n = 8), as a treatment (day 28 to day 38; n = 10) or as continuous exposure (day 0 to day 38; n = 10). The maximum eosinophil count was significantly greater in the oesophagus of sensitized mice challenged with Af extract than in the oesophagus of non-sensitized mice. Interestingly, sensitized mice receiving NCC 2287 from day 28 to 38 had significantly less oesophageal eosinophilia than the non-supplemented sensitized group. However, there was no significant effect on oesophageal eosinophilia when the NCC 2287 strain was administered as a preventive measure or when it was given continuously, throughout the study.

This study demonstrates that the time frame of supplementation is fundamental: the beneficial effect of L. lactis NCC 2287 was only observed when it was administered as a treatment. Altogether, the data suggest that L. lactis NCC 2287 may be an exciting candidate for reducing EoE inflammation.

B. lactis NCC 2818 and L. lactis NCC 2287 both induce an increase of IL-10 in a Th2-skewed PBMC model, suggesting a possible immunoregulatory effect of these strains (76–78). However, in the same model, L. lactis NCC 2287 is more potent at reducing IL-5 levels and a stronger inducer of IFN-c than B. lactis NCC 2818 (36). It seems debatable the evaluation of the predictive value of these assays in human disease.

Probiotics seem to stabilize IL10 mRNA expression and to dysregulate microRNAs in human monocyte (78). These results suggest that other epigenetic mechanisms could explain the effect observed in the oesophagus and that the understanding of probiotic impact is at the beginning. Akei et al. have demonstrated that oesophageal eosinophilia is IL-5-dependent (79).

The balancing impact of NCC 2287 on IL-5 expression seen in Th2-slanted PBMCs (75) and an ovalbumin/cholera toxin-induced food hypersensitivity model (74) may somewhat clarify the abatement in oesophageal eosinophilia seen with NCC 2287 treatment in this study.

These results suggest that L. lactis NCC 2287 may lead to a steady decrease in gastrointestinal Th2 inflammation independently of the level of antigen sensitization in experimental allergy models.

Previous studies have highlighted the noticeable link between oesophageal and lung inflammation (45). Furthermore, 14–70% of EoE patients also presented with asthma (1, 46). The effect of L. lactis NCC 2287 on lung eosinophilia and oesophageal eosinophilia could be referred to a common consequence of the probiotic on eosinophil recruitment. Nevertheless, oral supplementation with B. lactis NCC 2818 had no effect on EoE, although it significantly reduced bronchoalveolar eosinophilia.

Their data suggest that a decrease in eosinophils in the lung is not concomitant with a significant decline in oesophageal eosinophilia. These outcomes are to be expected as various preclinical examinations have indicated that lung eosinophilia can diminish with probiotic supplementation (80, 81) or auxiliary to changes in the gut microbiota (82, 83).

Probiotics could vary in their ability to prevent or deal with the allergic response.

The proof in the animal model recommends that specific probiotics might be gainful in lessening oesophageal eosinophilic inflammation.

Instead, probiotics seem to permit a more comprehensive approach which may restore and maintain homeostasis in humans.

**Figure 1:**
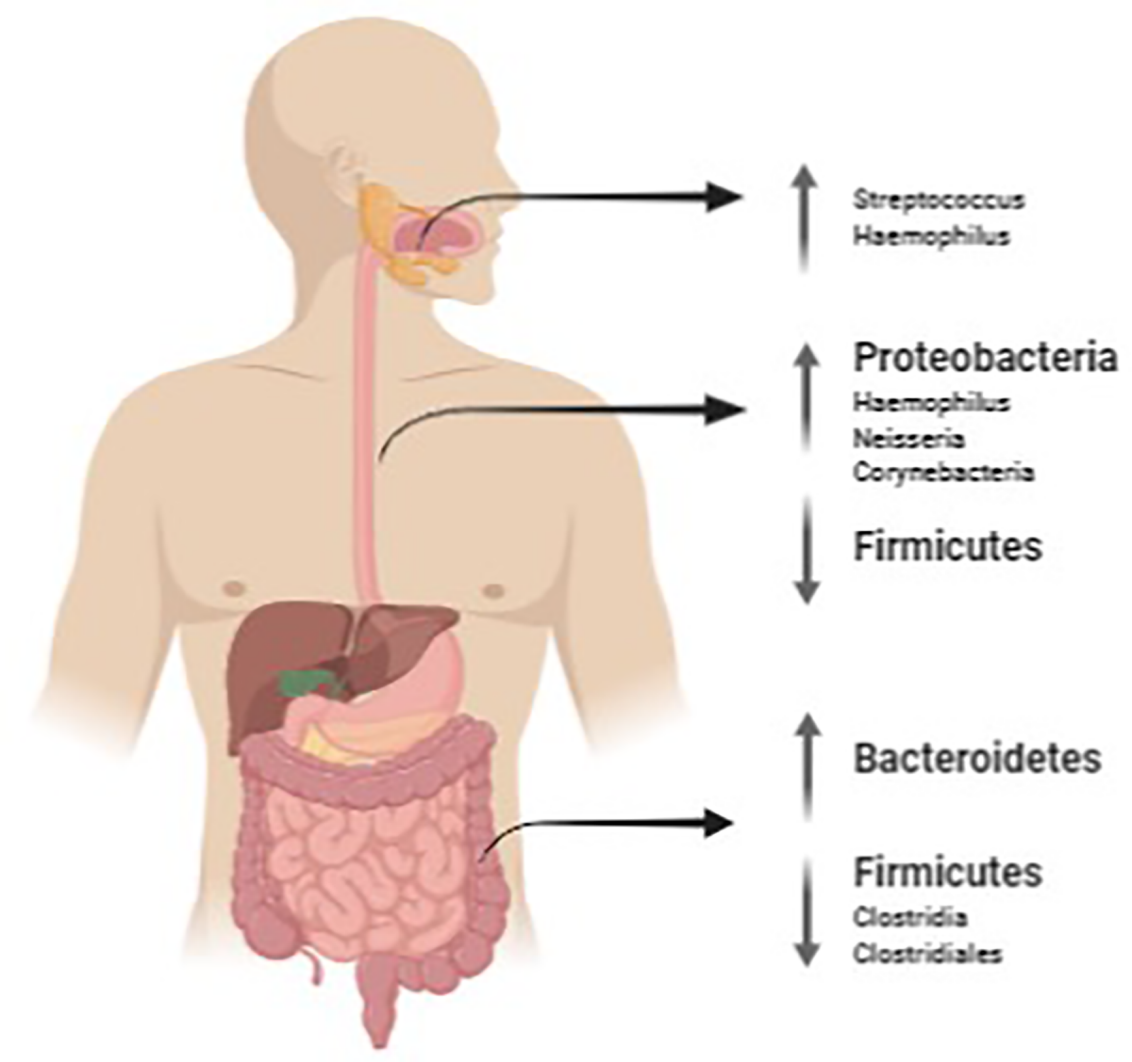
Salivary, oesophageal and gut microbiota mutations in EoE. The image was created with Biorender.com.

**Figure 1** summarizes the main findings of microbiota found in EoE at oral, esophageal and intestinal level.

## Conclusion

In conclusion, the oesophageal mucosa hosts a resident microbiota and there is evidence that it may change in the presence of EoE with an increase of bacterial load (56, 67) and with Streptococcus as recurrent taxa (67, 68) and with Haemophilus as possible marker of disease activity in different studies (56, 68).

It is not yet clear what is the cause-effect link that regulates these changes.

It is therefore not yet possible to imagine possible rational interventions for modulating the esophageal microbiota.

It therefore appears essential to carry out immunological studies that clarify this phenomenon and that allow to hypothesize also possible alternative therapies for EoE.

## Author Contributions

MM, AA, and AF conceived the review and research method of bibliographic sources. MM and RT performed the research, the analysis and the selection of the sources. MM and AA wrote the first draft of the manuscript. CR, FR, and PA performed the critical analysis of the sources and the final revision of the manuscript. All authors contributed to the article and approved the submitted version.

## Funding

The Scientific Direction of Bambino Gesù Children's Hospital approved and contributed to the payment of the publication fee of this scientific contribution.

## Conflict of Interest

The authors declare that the research was conducted in the absence of any commercial or financial relationships that could be construed as a potential conflict of interest.
